# Love, Pride and Worry: Grandparents’ Emotions in Daily Interactions with Their Grandchildren

**DOI:** 10.1093/geroni/igaf122.254

**Published:** 2025-12-31

**Authors:** Flavia Chereches, Yvonne Brehmer, Gabriel Olaru, Nicola Ballhausen

**Affiliations:** Tilburg University, Tilburg, Noord-Brabant, Netherlands; Tilburg University, Tilburg, Noord-Brabant, Netherlands; Tilburg University, Tilburg, Noord-Brabant, Netherlands; Tilburg University, Tilburg, Noord-Brabant, Netherlands

## Abstract

Past research investigating the impact of grandchild contact on grandparents’ well-being showed mixed results. Some studies reported benefits, while others revealed no effects or even negative consequences of grandchild contact for grandparents’ well-being. However, most of this research was cross-sectional or relied on data with large time gaps between assessments. Consequently, the short-term experiences of grandparenting remain unclear. We aimed to investigate how contact with grandchildren impacts grandparents’ positive and negative emotions in their daily lives, comparing this contact to interactions with others and time spent with a partner or alone. Using snippets of ambient sound recordings, we investigated if grandparents use more positive and less negative emotion-related words with grandchildren than with others. Our study involved 126 grandparents from the “Daily Experiences and Wellbeing study” followed over 5 days (6 assessments/day). Applying multilevel structural equation modeling, we found that grandchild contact was associated with stronger positive affect, specifically more love and pride, greater pleasantness, and more positive word use compared to interactions with others and being alone. However, grandparents also reported feeling sadder and more worried when being with their grandchildren, although these effects were comparatively weaker. Integrating multiple assessment methods (self-report and objective sound recordings), our findings show that while grandchild contact evokes a mix of emotions, increases in specific positive emotions within daily life are unique for interaction with grandchildren as compared to interactions with other individuals.

